# A Transgenic Mouse Line Expressing Cre Recombinase in Undifferentiated Postmitotic Mouse Retinal Bipolar Cell Precursors

**DOI:** 10.1371/journal.pone.0027145

**Published:** 2011-10-31

**Authors:** Philip E. B. Nickerson, Kara Ronellenfitch, Jason McEwan, Howard Kim, Roderick R. McInnes, Robert L. Chow

**Affiliations:** 1 Department of Biology, University of Victoria, Victoria, British Columbia, Canada; 2 Hospital for Sick Children, University of Toronto, Toronto, Ontario, Canada; 3 Lady Davis Research Institute, Department of Biochemistry, McGill University, Montreal, Quebec, Canada; Dalhousie University, Canada

## Abstract

Approaches for manipulating cell type-specific gene expression during development depend on the identification of novel genetic tools. Here, we report the generation of a transgenic mouse line that utilizes *Vsx2* upstream sequences to direct Cre recombinase to developing retinal bipolar cells. In contrast to the endogenous *Vsx2* expression pattern, transgene expression was not detected in proliferating retinal progenitor cells and was restricted to post-mitotic bipolar cells. Cre immunolabeling was detected in rod bipolar cells and a subset of ON and OFF cone bipolar cells. Expression was first observed at postnatal day 3 and was detectable between 24 hours and 36 hours after the last S-phase of the cell cycle. The appearance of Cre-immunolabeled cells preceded the expression of bipolar cell type-specific markers such as PKCα and Cabp5 suggesting that transgene expression is initiated prior to terminal differentiation. In the presence of a constitutive conditional reporter transgene, reporter fluorescence was detected in Cre-expressing bipolar cells in the mature retina as expected, but was also observed in Cre-negative Type 2 bipolar cells and occasionally in Cre-negative photoreceptor cells. Together these findings reveal a new transgenic tool for directing gene expression to post-mitotic retinal precursors that are mostly committed to a bipolar cell fate.

## Introduction

The successful progression of retinal development requires that complementary mechanisms of self-renewal and cell differentiation be finely regulated within retinal progenitor cells (RPCs). A variety of intrinsic, trans-activating proteins regulate this process and include members of the homeodomain family of transcription factors.

The Vsx2/Chx10 (herein referred to as Vsx2) transcription factor is a highly conserved paired-like homeodomain protein that is expressed in a number of developing central nervous system structures, including spinal cord, ventral hindbrain, and retina [1]. Essential functions of *Vsx2* orthologues have been demonstrated in species ranging from *C. elegans* [2], teleost [3], [4], [5], chick [6] and humans [7]. *Vsx2* is expressed in RPCs engaged in the cell cycle, and is retained in bipolar interneurons [8], and a subset of Müller glia [9]. *Vsx2* loss-of-function mutations lead to microphthalmia in mice [8] and humans [7]. In addition, there is a specific loss in the specification of bipolar interneurons in the mouse *Vsx2* loss-of-function mutant, which highlights the evolutionary conserved role of *Vsx2* in sensory interneuron development [8], [10], [11]. Recent evidence has revealed that *Vsx2* function shifts from that of cell cycle maintenance in early phases of RPC activity, to that of bipolar cell specification and repression of photoreceptor production during later phases of retinogenesis [12]. The highly conserved and pleiotropic function of the *Vsx2* gene, as well as its requirement for human ocular development make it a strong focus of retinal research.

A number of studies have examined the promoter sequences and upstream regulatory elements of *Vsx2* [9], [13], [14]. One such study utilized a bacterial artificial chromosome (BAC) harbouring ∼100 kb *Vsx2* upstream sequence to generate transgenic reporter mice [9]. Reporter expression in this transgenic mouse was mosaic, but recapitulated putative Vsx2 protein localization in RPCs, differentiated bipolar cells and Müller glia. More detailed analyses of upstream regulatory elements identified a 22 bp sequence located within a 3 kb upstream region required for *Vsx2* expression postnatally [13], and a 164 bp sequence located ∼19 kb upstream of the *Vsx2* start site that is sufficient to drive bipolar cell-specific expression [15]. Therefore it appears that distinct *Vsx2* upstream regulatory sequences are able to direct accurate retinal expression of *Vsx2* in a spatiotemporal context. In this report, we describe the generation of a novel transgenic mouse line utilizing *Vsx2* upstream sequences to direct the expression of Cre recombinase. *Vsx2-5.3-PRE-Cre* mice exhibit highly restricted Cre expression that partially overlaps with the temporal and cell type-specific expression of putative *Vsx2*. Birth dating experiments demonstrated that Cre expression is restricted to a large subset of postmitotic bipolar cells and not detectable in RPCs or Müller glia. This transgene also identifies what appears to be transient or below detectable levels of activity of the *Vsx2* transgene in either bipolar/photoreceptor precursors or photoreceptors. Together, these data reveal a novel transgenic tool that can be used for the conditional targeting of post-mitotic bipolar cells.

## Materials and Methods

### Generation of *Vsx2-5.3-PRE-Cre* mice

A 5.3 kb region upstream of murine *Vsx2* that extends into the 5′ UTR region up to the *Vsx2* start codon was cloned into the *Not*I and *Sac*II sites of pBluescript (Stratagene/Agilent, Santa Clara, CA). A 2 kb putative retinal enhancer (PRE) region within the 5′ breakpoint in the *Vsx2 or-2J* (*ocular retardation-2J*) allele and containing the 164 bp bipolar enhancer region [15] was amplified and cloned 5′ of the 5.3 kb upstream sequence. Cre recombinase was cloned downstream of the 5.3 kb *Vsx2* region and was followed by an SV40 poly adenylation sequence. Transgenic mice were generated in a C57×C3H strain background offspring were identified by PCR genotyping of the Cre insert using either ear clip or tail genomic DNA preparations. Primers sequences used were as follows: Cre-forward–5′- CCC ATG GTC TTC TTC TGC AT – 3′; Cre-reverse – 5′- CCA TGA GTG AAC GAA CCT GG – 3′.

### Animal Husbandry and Breeding

All experimental procedures performed on mice as well as housing of mice was done with the approval of the University of Victoria Animal Care Committee (Protocol 2008-013) following standards described by the Canadian Council for Animal Care. *Vsx2-5.3-PRE-Cre* mice were maintained by breeding founder mice with non-transgenic background strains (129S1 or CD1, The Jackson Laboratory, Bar Harbor ME). *mGluR6:NLS-LacZ* mice express a nuclear localized version of β-galactosidase under the control of the *mGluR6* upstream sequence [16]. *Gt(ROSA)26Sor^tm9(CAG-tdTomato)Hze^* mice (The Jackson Laboratory, Stock number 007914) have a *loxP*-flanked STOP cassette preventing transcription of a CAG promoter-driven red fluorescent protein variant (tdTomato), and was used as a Cre reporter strain.

### Metabolic labeling of proliferating cells and their progeny

To label cells during the S-phase of mitosis, a single intraperitoneal injection of 5′-chloro 2-deoxy-uridine (CldU–46 mg/kg, Sigma-Aldrich, Ontario, Canada) was performed on postnatal pups ranging from P0-P6. This dosage and regimen of administration has been shown to consistently label proliferating cells in the central nervous system [17], [18], [19]. Preliminary immunocytochemical assessment confirmed that CldU labeling was restricted to the nucleus using TO-PRO® -3 iodide (1∶1000-T-3605, Molecular Probes, Oregon, USA).

### Tissue processing and Immunolabeling

Adult mice were anesthetized and euthanized by cervical dislocation. Eyes were enucleated, washed in chilled phosphate buffered saline PBS (pH 7.4) and were fixed either by emersion in chilled 4% paraformaldehyde (PFA) (Electron Microscopy Sciences, Hatfield, PA) for 25 minutes or overnight at 4 deg C. Fixed eyes were washed in PBS and cryoprotected in 30% sucrose for 18 hours. Following block embedding, serial cryostat sectioning was performed at a thickness of 12 µm. For immunolabeling, mounted sections were washed in phosphate buffered saline (PBS), permeabilized in 1% Triton-X 100 for 30 minutes at room temperature, and incubated with in primary antibody diluted in PBS (Table 1). In the case of CldU immunolabeling, sections were placed in 2N HCL for 45 minutes at room temperature prior to permeabilization. Sections were then rinsed and incubated with Alexa-conjugated fluorescent secondary antibodies (1∶500-Invitrogen, ON, Canada) for 1 hour at room temperature, followed by washing in PBS and mounting in Immuno-mount (Shandon, PA, USA).

**Table 1:** List of Antibodies.

Antigen	Antiserum	Source	Working dilution
Chx10/Vsx2	sheep anti-Chx10	Exalpha Biologicals (X1180P)	1∶500
Vsx1	rabbit anti-Vsx1	RL Chow	1∶100
Calbindin D-28k	rabbit anti-Calbindin-D-28K	Sigma (C 2724)	1∶1000
Cabp5	rabbit anti-Cabp5	F. Haeseleer, Department of Ophthalmology, Seattle, WA	1∶500
β-Gal	rabbit anti-β-Gal	ICN, Aurora, OH (55976)	1∶20,000
PKCα	rabbit anti-PKCα	Sigma (P4334)	1∶10,000
Bhlhb5	goat anti-β3(E17)	Santa Cruz (sc-6045)	1∶1000
PKA RIIβ	mouse anti- PKA RIIβ	BD science (612550)	1∶3,000
Cre recombinase	mouse anti Cre	Covance (MMS-106)	1∶1000
Cre recombinase	rabbit anti-Cre	Covance (PRB-106c)	1∶2000
BrdU (CldU)	rat anti-BrdU	Accurate Chemical & Scientific	1∶500

### Confocal Microscopy

Immunolabeled sections were imaged using either a Nikon Eclipse TE-2000-U or a Zeiss LSM700 confocal microscope equipped with APO TIRF 1.49 oil/DIC objective lenses (20-63X magnification). Pinhole diameters were maintained at 1 AU and laser outputs were optimized for individual staining intensities. Emission spectra were matched to secondary antibodies and tdTomato spectral characteristics. Orthogonal analysis was used to ensure co-localization of contrasting subcellular staining domains. Images were cropped and processed for brightness using Adobe Photoshop CS3.

## Results

### Expression of the *Vsx2-5.3-PRE-Cre* transgene in mouse bipolar cells

**Figure 1:**
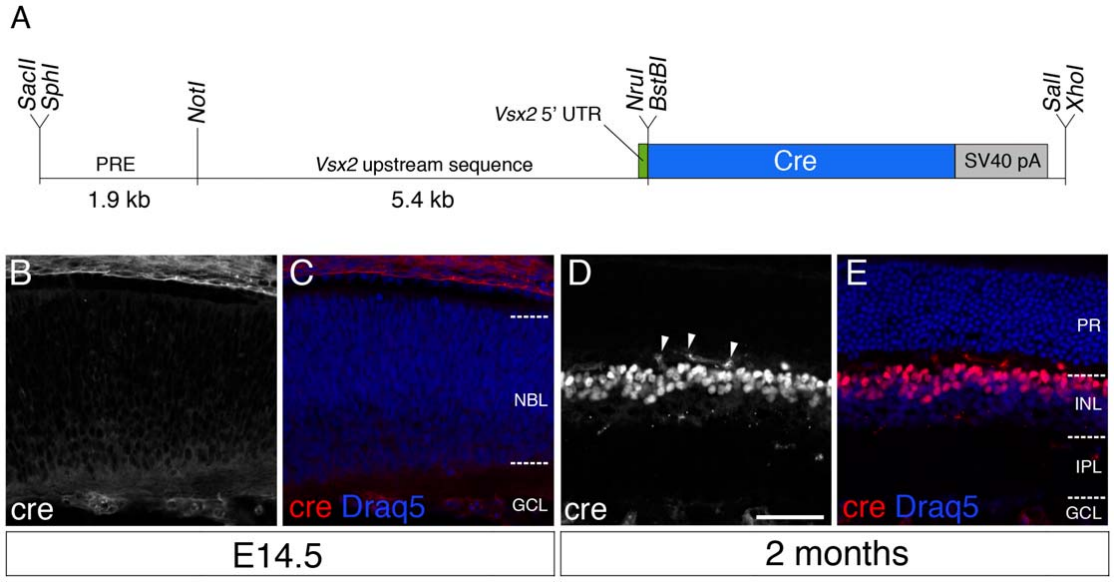
*Vsx2-5.3-PRE-Cre* is expressed in the postnatal and adult retinal inner nuclear layer. (A) The *pLac-Vsx5.3-PRE* transgene construct contains a 1.9 kb *Vsx2* upstream conserved region (UCR), cloned 5′ of a 5.4 kb genomic fragment containing the 5′UTR of *Vsx2* and followed by a Cre recombinase encoding fragment. Abbreviations *UTR*: untranslated region; *SV40 pA*: SV40 polyadenylation signal. Cre recombinase immunolabeling is not detected in either the neuroblastic layer (NBL) or ganglion cell layer (GCL) of E14.5 retinas (B, C), but is detected in the outer portion of the inner nuclear layer (INL) in adult mice (D, E). Arrowheads in (D) indicate non-specific labelling of blood vessels by mouse anti-Cre antibody. Scale bar  = 50 µm.

The *Vsx2-5.3-PRE-Cre* transgene was generated such that a 5.3 kb upstream region of *Vsx2* plus a 1.9 kb region located 18 kb upstream of the *Vsx2* start codon was placed upstream of a Cre-expressing cDNA cassette (Figure 1A). The 1.9 kb *Vsx2* upstream fragment was originally identified as a potential retinal enhancer (PRE) as it lies within the breakpoint of the *ocular retardation 2J* mutation within a non-coding region that is conserved between human and mouse (M. Burmeister, pers. comm.). Six lines of *Vsx2-5.3-PRE-Cre* mice were generated, all of which carried the Cre transgene as identified by PCR genotyping (Table 2). Expression of Cre recombinase, as determined by Cre immunolabeling was detected in 4 of these lines (Table 2). In contrast to the normal expression of *Vsx2* in retinal progenitor cells at E14.5 [1], Cre labeling was not detectable in retinas from E14.5 *Vsx2-5.3-PRE-Cre* mice (Figure 1B,C). Examination of adult tissue from all lines revealed that *Vsx2-5.3-PRE-Cre* retinas exhibited normal thickness and lamination, and contained robust nuclear localized Cre immunolabeling within the inner nuclear layer (Figure 1D,E). The location of Cre-labeling was consistent with the pan-bipolar expression of Vsx2, and did not extend into the ciliary epithelium or other non-neuroretinal structures (not shown).

**Table 2:** Summary of *Vsx2-5.3-PRE-Cre* transgenic mouse lines.

Founder	Cre immunolabeling*	Conditional reporter expression**
2245	−	−
2690	+	+
2695	−	−
2697	+	+
2700	+	+
2717	+	+

*“+” indicates Cre immunolabeling restricted to postmitotic presumptive bipolar cells and/or mature retinal bipolar cells

**“+” indicates reporter expression in the mature retina in bipolar cells and a subset of photoreceptor cells

**Figure 2 pone-0027145-g002:**
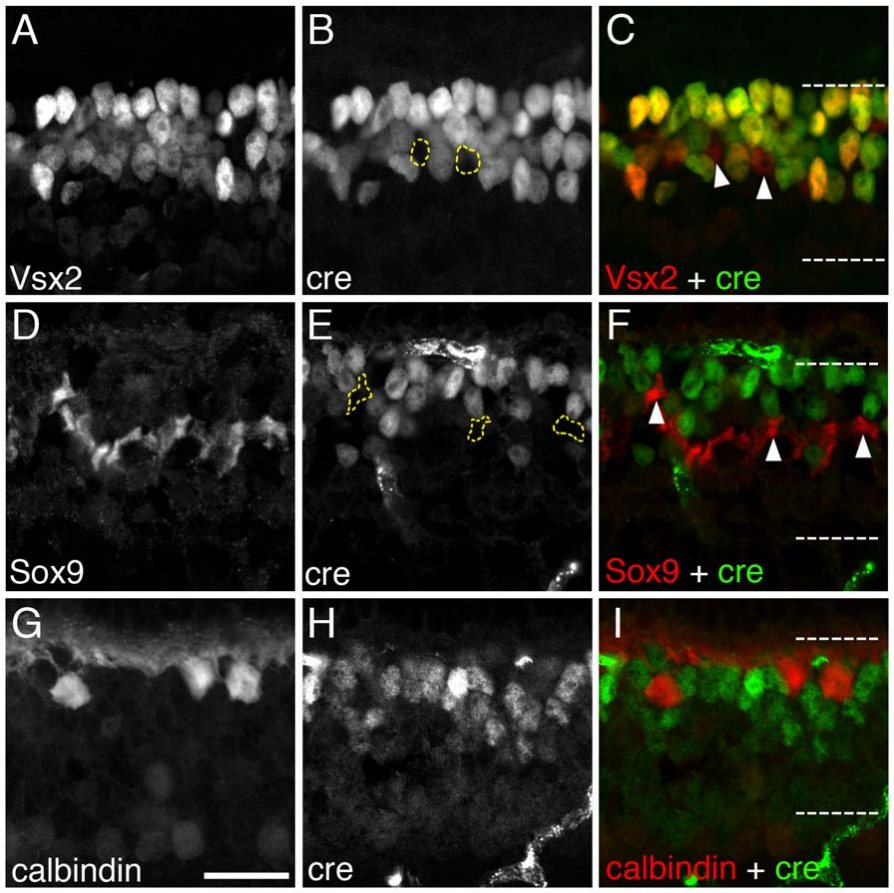
*Vsx2-5.3-PRE-Cre* is specifically localized to Vsx2-expressing neurons of the inner nuclear layer in the mature retina. (A–C) The vast majority of Vsx2-labeled nuclei (A) in the inner nuclear layer (boundaries indicated by broken lines) co-label for Cre (B). Arrowheads in (C) indicate Vsx2-positive/Cre-negative nuclei outlined in (B). (D–F) Sox9-positive Müller glial nuclei (D) do not express Cre. Arrowheads (F) show examples of Cre-negative/Sox9-positive nuclei outlined in (E). (G–I) Horizontal cells labelled with Calbindin-D28k (G) do not label for Cre (H). Sections are from >6 week old mice. Scale bar  = 10 µm.

To verify that the *Vsx2-5.3-PRE-Cre*transgene was expressed in bipolar cells, we next examined Cre transgene expression in the mature retina double-immunolabeled for Cre and Vsx2. Co-immunolabeling of Cre with Vsx2 verified that 80% of Vsx2-postive nuclei located in the outer inner nuclear layer were Cre-positive, whereas 100% of Cre labeled nuclei co-labeled with Vsx2 (Figure 2A–C). As Vsx2 is also weakly expressed in Müller glia [9], we double-labeled retinas with the Müller marker Sox9 [20], but were unable to detect co-immunolabeling (Figure 2D–F). Similarly, co-immunolabeling of sections with Cre and the horizontal and amacrine cell marker, Calbindin D-28k [21], verified that Cre was not detectable in these other inner nuclear layer interneurons (Figure 2G–I). These results show that *Vsx2-5.3-PRE-Cre* is not expressed at detectable levels in the embryonic retina, and is restricted to a large subset of Vsx2-expressing bipolar cells in the mature retina.

### *Vsx2-5.3-PRE-Cre* is expressed in a large subset of adult bipolar neurons

**Figure 3:**
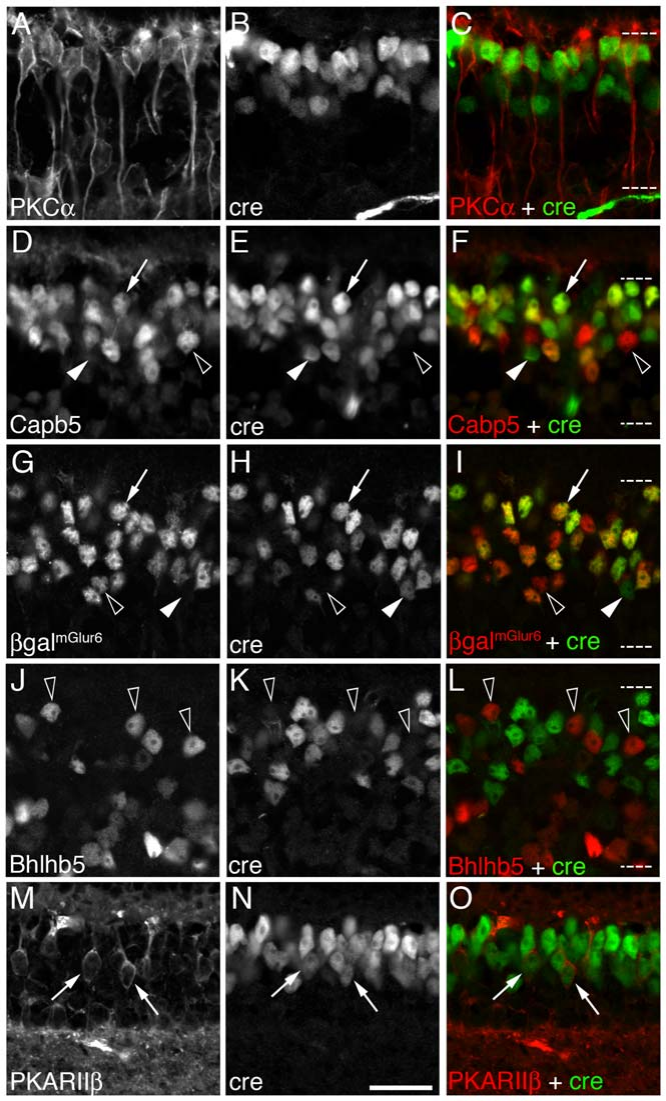
*Vsx2-5.3-PRE-Cre* is expressed in a large subset of bipolar neurons in the adult retina. Immunolabeling for Cre-recombinase under control by the *Vsx2-5.3-PRE* transgene is localized in nearly all PKCα expressing rod bipolar cells (A–C); a large subset of CaBp5-expressing Type-III a/b, Type-V ON, or rod bipolar cells (D–F); *mGlur6-lacZ* expressing ON bipolar cells (G–I); and a large subset of PKARIIβ-expressing Type-IIb and OFF bipolar neurons (J–L). Bhlhb5 expressing Type-II OFF bipolar cells do not express Vsx2-5.3-PRE-Cre (M–O). Arrows indicate co-localized cells; solid arrowheads indicate Cre-only cells; open arrowheads indicate Cre-negative cells. Scale bar (N)  = 10 µm.

To determine the cellular subtype specificity of the *Vsx2-5.3-PRE-Cre* transgene expression, we next evaluated Cre immunolabeling in combination with panel of bipolar cell markers (Table 1). At least 11 subtypes of bipolar interneurons can be identified based on their morphology and distinct gene expression patterns [22], [23], [24], [25]. *Vsx2-5.3-PRE-Cre* co-localized with nearly all PKCα-expressing rod bipolar cells (Figure 3A–C) and with approximately 50% cells labeled with Cabp5, a marker of Type 3 OFF, Type 5 ON and rod bipolar cells [23](Figure 3D–F). To determine whether *Vsx2-5.3-PRE-Cre* is absent from all ON bipolar cells, we crossed *Vsx2-5.3-PRE-Cre* mice with a transgenic strain of mice expressing a nuclear-localized version of β-galactosidase under the control of the *mGluR6* 9.5 kb upstream region [16], which selectively directs expression to all ON type bipolar cells [26]. *Vsx2-5.3-PRE-Cre* was localized within a large subset of *mGluR6*:β-galactosidase immunolabeled-expressing cells (Figure 3G–I, arrows) although not all *mGluR6*:β-galactosidase labeled cells were co-labeled for Cre (Figure 3G–I, black arrowheads). In addition, some Cre-labeled cells did not label for *mGluR6*:β-galactosidase (Figure 3G–I, white arrowheads) suggesting that they were OFF bipolar cell types. We found no examples of Cre labeling in Bhlhb5-expressing Type 2 OFF bipolar cells (Figure 3J–L), however we did observe co-labeling with PKARIIβ (Figure 3M–O, arrows), which labels Type 3b OFF bipolar cells[24]. These observations indicate that *Vsx2-5.3-PRE-Cre* is detectable in a broad subset of rod, OFF and ON bipolar cells, but is not detectable in Type 2 OFF bipolar cells and a subset of ON bipolar cells.

### Onset of *Vsx2-5.3-PRE-Cre* transgene expression

**Figure 4:**
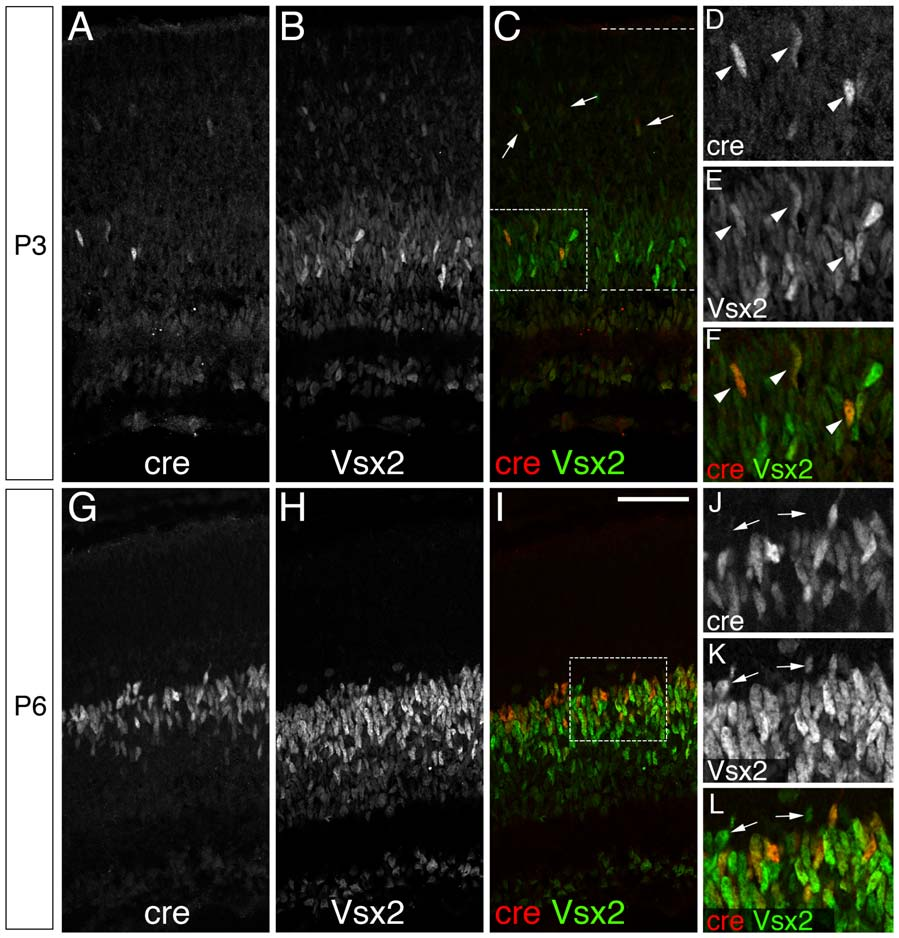
*Vsx2-5.3-PRE-Cre* is up-regulated postnatally in Vsx2-expressing cells. (A–D) P3 marks the earliest age at which Vsx2-5.3-PRE-Cre can be detected by immunofluorescence. Strong, Cre-immunolabeled nuclei (A) co-label with Vsx2 (B) within the inner region of the Vsx2-positive neuroblastic layer (demarcated with dashed lines in C). Inset (D–F) shows high magnification view of examples of Cre/Vsx2 double-labeled nuclei (arrowheads) within the NBL (box in (C)). (G–I) By P6, a robust up-regulation of Vsx2-5.3-PRE-Cre is evident in Vsx2-expressing cells located throughout the NBL, although the majority of these cells localize at the apical margin of the NBL. Insets (J–L) show a high magnification view of the apical NBL. The presence of Cre-negative/Vsx2-positive nuclei located at the apical NBL boundary (arrows) may represent a newly postmitotic (G1) cell arriving to the Cre-expressing layer.

**Figure 5:**
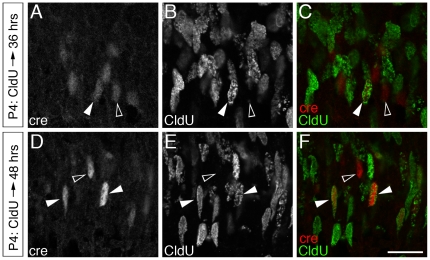
*Vsx2-5.3-PRE-Cre* is up-regulated postmitotically, and corresponds to early bipolar cell differentiation. (A–C) Retinas pulsed with CldU at P4 showed examples of Cre/CldU co-localization (solid arrowheads) at 36 h. This is in contrast to only rare examples detected at 24 h (not shown). The presence of adjacent, Cre-positive/CldU-negative nuclei (open arrowheads) indicate that the timing of Cre onset of expression is tightly regulated. (D–F) By 48 h, Cre/CldU cells were clearly evident at high frequency (G–O).

In contrast to the endogenous expression pattern of *Vsx2* [1], expression of the *Vsx2-5.3-PRE-Cre* transgene was not detected in the embryonic retina. Weak Cre immunolabeling was first observed in the central portion of the retina at P3 (Figure 4A–F). Cre-positive nuclei were highly localized in the developing neuroblastic layer (NBL), and co-immunolabeled with Vsx2 (Figure 4 A–F). Although the majority of Cre-positive nuclei resided within the Vsx2-rich inner neuroblastic layer (iNBL) (Figure 4D–F), a small number of Cre-immunolabeled nuclei were evident in the apical retina that also immunolabeled for Vsx2 (Figure 4C - arrows). By P6, robust Cre immunolabeling was present throughout the extent of the central and peripheral retina, and was predominantly distributed within the outer portion of the iNBL (Figure 4G–L). Since Vsx2-expressing cells at P3 and P6 may include both proliferating and postmitotic cells, we pulsed P4 mice with the thymidine-analogue CldU to metabolically label cells in S-phase of the cell cycle in order to determine whether any of the Cre-immunolabeled cells were mitotically active. Retinas were fixed for histology at 12-hour intervals after CldU injection. Only a single example of Cre/CldU co-labeling was captured in the central retina at 24 hours post injection (not shown; n = 3 animals), but by 36 hours, co-labeling of CldU-positive cells with faint Cre-immunolabeled cells was clearly evident in the central portion of the retina (Figure 5A–C). By 48 hours after injection, the population of Cre/CldU double labeled nuclei was highly enriched in the iNBL (Figure 5D–F).

**Figure 6:**
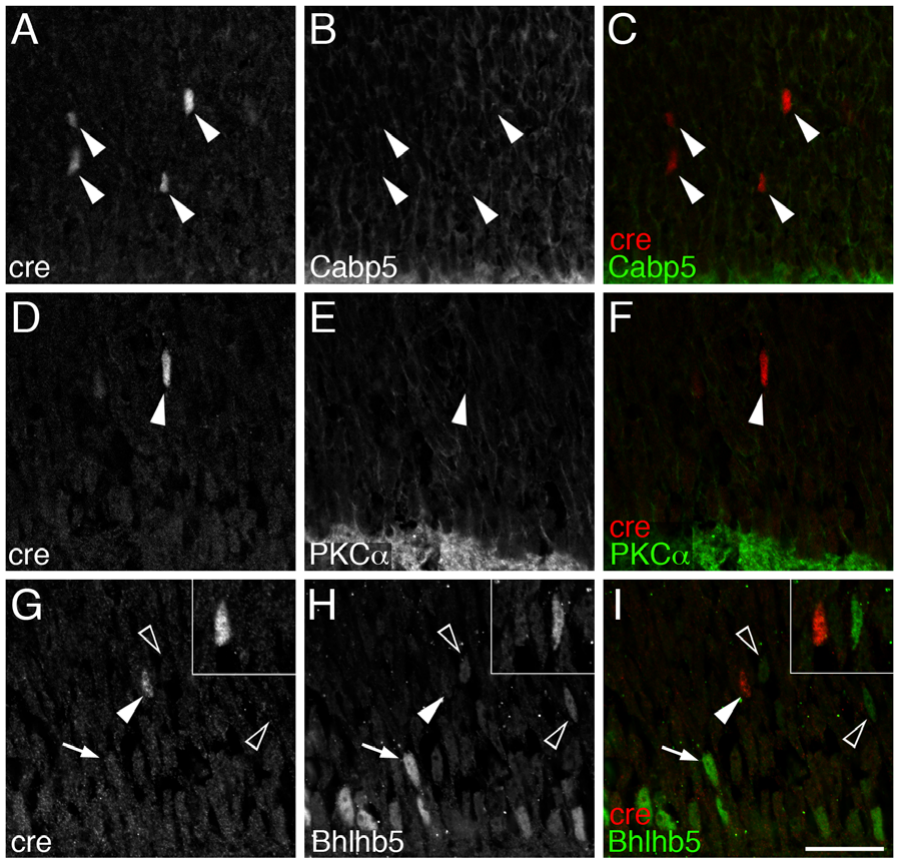
Cre expression precedes expression of the mature bipolar markers PKCα and Cabp5. At postnatal day 3 (P3) Cre-positive nuclei do not co-localize to Cabp5 (A–C), PKCα (D–F), or with the early bipolar fate marker Bhlhb5 (M–O). Insets (G–I) are high magnification view of Cre-positive (solid arrowhead) and Cre-negative (open arrowhead) nuclei located apical to the Bhlhb5-positive layer (arrow for example). Scale bar (O)  = 20 µm.

Having established that the onset of Cre expression is first detectable between 24 and 48 hours after the last S-phase, we sought to identify whether Cre expression precedes the expression of bipolar fate and differentiation markers. In contrast to the robust *Vsx2-5.3-PRE-Cre* expression observed in adult Cabp5 and PKCα expressing bipolar cells (Figure 3), Cre immunolabeling appeared before the onset of Cabp5 (Figure 6A–C) or PKCα (Figure 6D–F) bipolar cell immunolabeling in P3 retinas. Consistent with an absence of co-localization with Bhlhb5 in adult (Figure 3), no co-labeling was evident at P3 (Figure 6 G–I), despite the presence of Cre-positive and Bhlhb5-positive nuclei located apically of Bhlhb5-expressing, putative amacrine cells (Figure 6G–I). It is unclear, however, whether the Bhlhb5-labeled cells residing apical of putative amacrine cells (e.g. Figure 6G–I, black arrowheads) label progenitor cells, newly born bipolar cells and/or amacrine cells. Together, these data indicate that *Vsx2-5.3-PRE-Cre* is expressed in newly postmitotic cells and precedes the expression of mature bipolar cell markers.

### Genetic lineage tracing reveals a sub-population of transient *Vsx2-5.3-PRE-Cre* expressing cells

**Figure 7:**
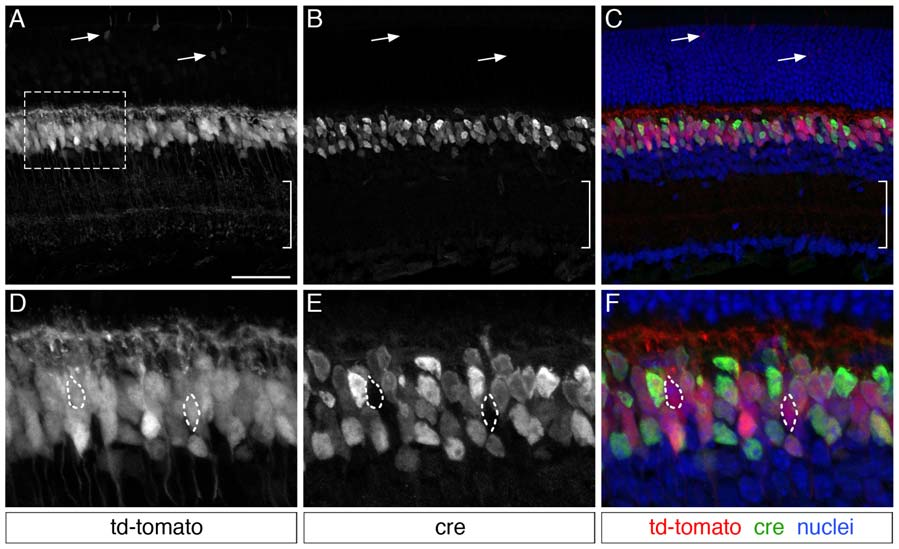
Genetic fate mapping of the *Vsx2-5.3-PRE-Cre* transgene using tdTomato conditional reporter mice. *Vsx2-5.3-PRE-Cre* mice were crossed with mice containing the *Gt(ROSA)26Sor^tm9(CAG-tdTomato)Hze^* transgene. Offspring in which Cre-mediated recombination events were present continually express tdTomato in those cells. (A–C) The vast majority of tdTomato-expressing cells in *Vsx2-5.3-PRE-Cre/CAG:cond-tdTomato* mice were located in the outer portion of the inner nuclear layer. The morphology of these cells was consistent with that of a bipolar fate, including terminal lamination in the inner plexiform layer (bracketed region). Immunolabeling for Cre revealed tdTomato-expressing cells in which Cre expression does not persist in the adult (open arrowheads). A second population of tdTomato expressing cells is also evident in the outer nuclear layer, and bear morphological resemblance to photoreceptors (arrows).

**Figure 8:**
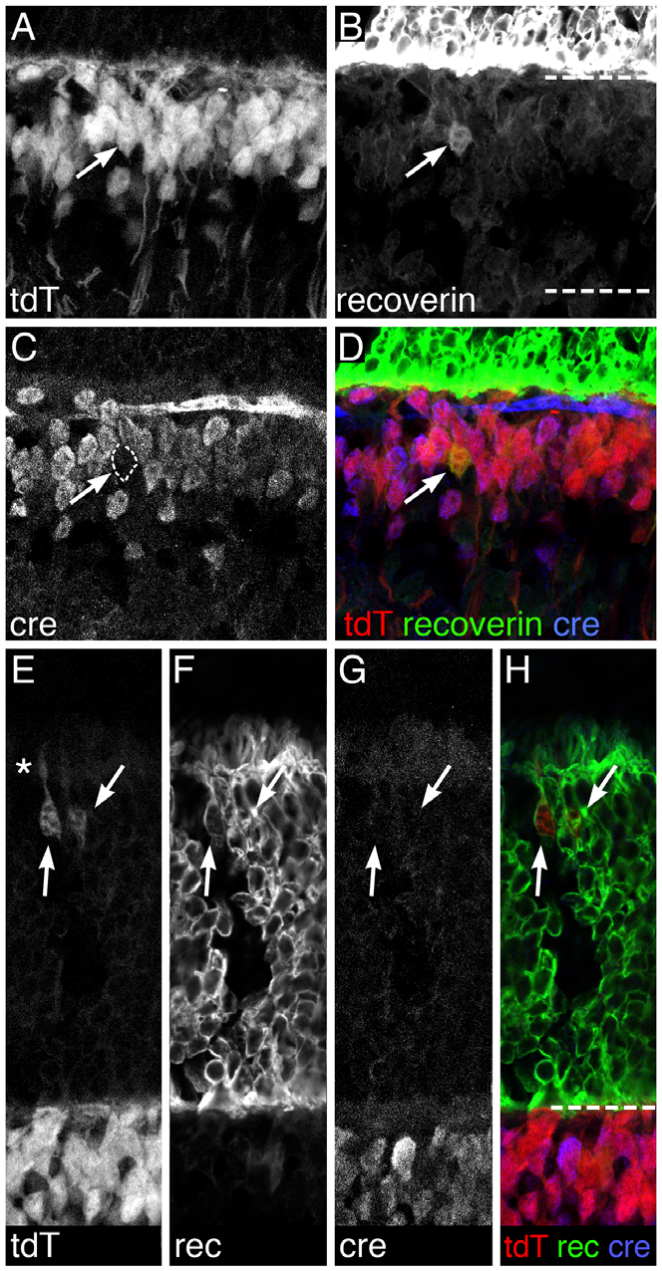
*Vsx2-5.3-PRE-Cre* expression in Type 2 bipolar cells and photoreceptors of the mature retina indicated by tdTomato conditional reporter expression. TdTomato fluorescence in >6 weeks old *Vsx2-5.3-PRE-Cre* mice harbouring the *Gt(ROSA)26Sor^tm9(CAG-tdTomato)Hze^* transgene did not co-label Type 2 bipolar cells immunolabeled for recoverin (A–D, arrow) or photoreceptors that were co-labeled for recoverin (E–H, arrows). The asterisk in (E) indicates a region of the tdT-fluorescing cell with photoreceptor outer segment morphology. The dashed lines in (B) and (H) indicate the boundaries of the inner nuclear layer.

Although we were unable to detect *Vsx2-5.3-PRE-Cre* immunolabeling in the embryonic retina (Figure 1), we next wanted to explore the possibility that the *Vsx2-5.3-PRE-Cre* transgene was expressed either transiently, or at sub-detectable levels during this period. To examine these possibilities, we utilized a Cre-sensitive conditional reporter transgenic mouse line. *Vsx2-5.3-PRE-Cre* mice were crossed to *Gt(ROSA)26Sor^tm9(CAG-tdTomato)Hze^* mice (abbreviated as *CAG:cond-tdTomato*)(Jackson Labs), which contain a targeted loxP flanked STOP codon which prevents tdTomato expression in cells not expressing Cre recombinase. TdTomato expression in adult *Vsx2-5.3-PRE-Cre/CAG:cond-tdTomato* retinas largely recapitulated the Cre immunolabeling expression pattern observed above (Figures 1–3). Specifically, tdTomato-positive cells were present in the outer tier of the INL, and exhibited morphological features of bipolar neurons including inner plexiform layer axon terminal patterning (Figure 7A–C, bracketed region). Cre immunolabeling of *Vsx2-5.3-PRE-Cre/CAG:cond-tdTomato* retinas revealed that the vast majority of tdTomato cells in the inner nuclear layer co-labeled with Cre, although examples of tdTomato-positive/Cre-negative cells were evident (e.g. outlined cells in Figure 7D–F). At least some of these cells were recoverin-positive Type 2 OFF bipolar cells (Figure 8A–D), consistent with the lack of Cre immunolabeling in putative Type 2 bipolar cells (Figure 3J–L). In addition to bipolar cell expression, a second, infrequent population of tdTomato-expressing photoreceptor cells was identified in the outer nuclear layer (Figure 7A–C, arrows). TdTomato-positive photoreceptors were more commonly observed in the retinal periphery and less frequently in the central retina (not shown). These cells co-labeled with the photoreceptor marker recoverin however, consistent with our findings above, Cre immunolabeling was undetectable in photoreceptor cells (Figure 8E–H). These genetic lineage tracing data demonstrate that the *Vsx2-5.3-PRE-Cre* Cre protein is functional, and that a Cre-sensitive reporter is able to faithfully recapitulate the *Vsx2-5.3-PRE-Cre* Cre immunolabeling pattern in bipolar cells.

## Discussion

**Figure 9:**
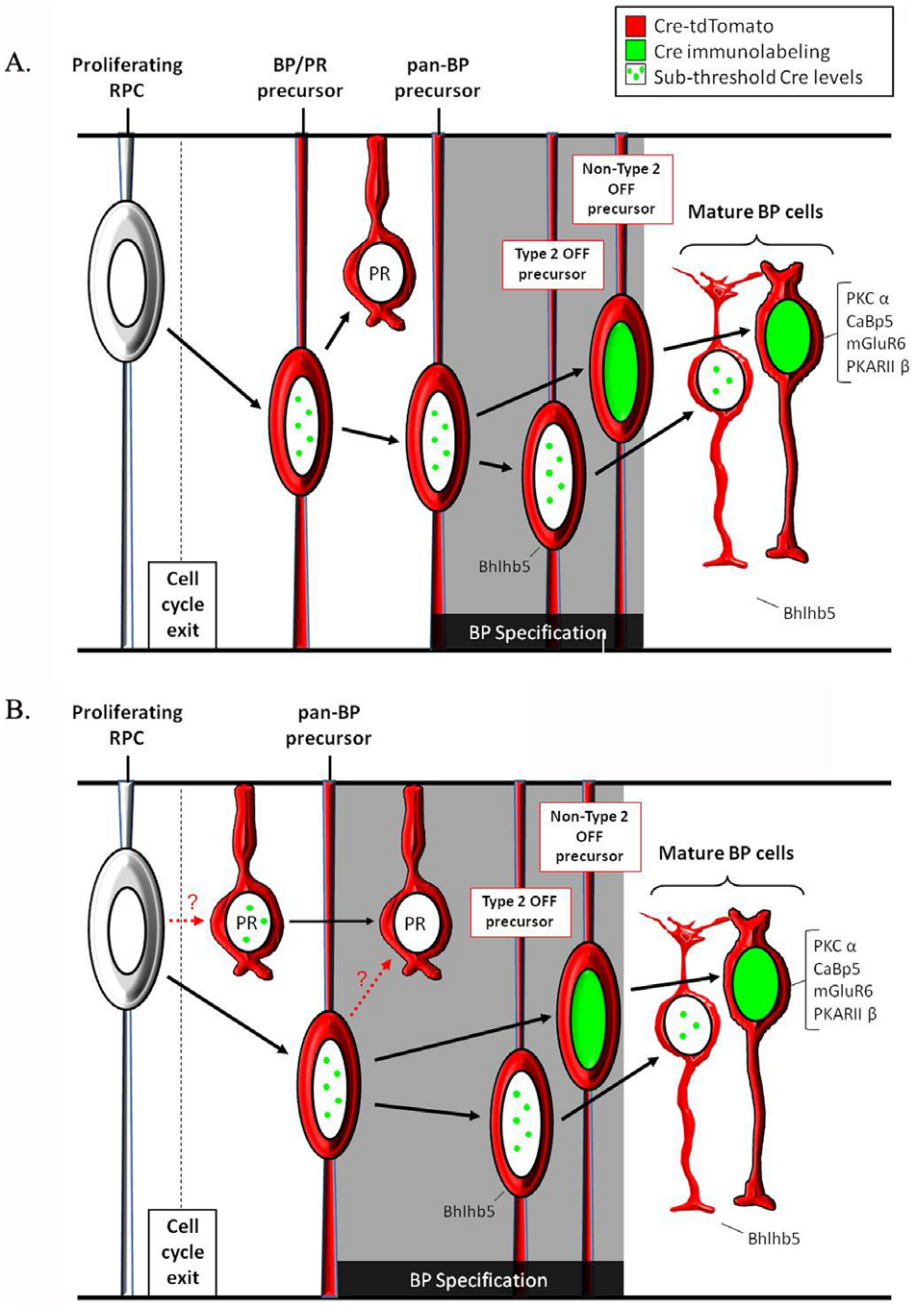
Summary of *Vsx2-5.3-PRE-Cre* expression in the developing retina. Although Cre immunolabeling is not evident in proliferating RPCs, genetic fate mapping identifies *Vsx2-5.3-PRE-Cre* activity in bipolar and photoreceptor cells. (A) Following bipolar cell specification, Cre immunolabeling is strongly up-regulated in all bipolar neuron subtypes, with the exception of blhlb5-positive Type 2 OFF cells. TdTomato-positive photoreceptors could be derived from postmitotic bipolar cell/photoreceptor cell precursors. (B) Alternatively, TdTomato expression may result from transient low-level Cre expression in photoreceptor cells derived from either RPCs, or from uncommitted bipolar cell precursors that switch their fate to that of photoreceptor cells.

Here we describe the development of a novel transgenic tool that can be used to target postmitotic bipolar interneurons using Cre recombinase. The *Vsx2-5.3-PRE-Cre* transgene is expressed in the majority of Vsx2-expressing bipolar subtypes, but is not detectable in Vsx2-expressing RPCs, Müller glia or non-neuroretinal structures. Using a conditional reporter approach, we also detected *Vsx2-5.3-PRE-Cre* transgene expression in a small subset of photoreceptors (summarized in Figure 9).

### Timing of Vsx2-5.3-PRE-Cre onset

*Vsx2-5.3-PRE-Cre* immunolabeling is first evident at postnatal day 3 and represents one of the earliest expressed bipolar cell markers reported to date. The onset of this expression, combined with the observation that Cre expression is first detected 24 hours after the last S-phase is consistent with our finding that bipolar cell birth in mice begins between postnatal days 1 and 2 (data not shown). *Vsx2-5.3-PRE-Cre* expression also precedes the expression of other bipolar cell-specific markers. For example, *Vsx1*, a *Vsx2* homologue that regulates terminal gene expression in differentiating Types 2, 3a and 7 bipolar cells [16], [27], [28], is first detectable in the retina at postnatal day 5. In addition, expression of the ON bipolar cell-specific gene *mGluR6* as well as the *mGluR6:β-gal* transgene reporter is first detectable at postnatal day 6 [26]. These findings suggest that *Vsx2-5.3-PRE-Cre* transgene expression defines an early population of undifferentiated bipolar cells (Figure 9A). Although the existence of an undifferentiated pan-bipolar cell type has yet to be demonstrated, the *Vsx2-5.3-PRE-Cre* transgene could serve as a useful tool for addressing this possibility.

### Comparison of *Vsx2-5.3-PRE-Cre* and *Vsx2 BAC* transgenic mice

In contrast to the postmitotic expression observed in *Vsx2-5.3-PRE-Cre* mice, the previously published *Vsx2 BAC* mice exhibit mosaic, Cre-mediated reporter expression in multipotent RPCs as early as E10 [9]. In the postnatal retina, alkaline phosphatase reporter expression in *Vsx2 BAC* mice is retained in postmitotic bipolar and Müller cells, and persists in the adult. Further characterization of *Vsx2* upstream regulatory sequences identified a number of retinal enhancer regions and cis-binding sites for *Vsx2* [13], [15]. Specifically, alkaline phosphatase reporter mice were generated using a 2.4 kb or 3.0 kb *Vsx2* upstream region, both of which faithfully recapitulate putative *Vsx2* retinal expression [13]. A 22 bp motif residing in the first 500 bp upstream region of *Vsx2* was necessary for expression in RPCs, and was identified as a POU factor (Brn-2 and Tst-1/SCIP) binding domain. The absence of RPC expression in *Vsx2-5.3-PRE-Cre* mice is interesting given that the *Vsx2-5.3-PRE-Cre* transgene includes the 3.0 kb *Vsx2* upstream RPC enhancer region. The lack of RPC expression in our transgenic mice might be due to repression of the RPC enhancer caused by the close proximity of the distal 2.5 kb region Putative Retinal Enhancer (PRE). This region contains the 164 bp region ∼19 kb upstream of the *Vsx2* start site that functions as a bipolar cell specific enhancer but has also been shown to function as a repressor in photoreceptor cells [15]. The lack of RPC expression in our transgene may also indicate the existence of a RPC repressor element within the 3.0 kb and 5.3 kb *Vsx2* upstream region. Alternatively, additional missing elements may be required may be required for RPC expression.

### *Vsx2-5.3-PRE-Cre* as a genetic tool for targeting specified bipolar cells and photoreceptor-competent precursors

The *Vsx2-5.3-PRE-Cre* transgene is expressed in the major bipolar cell sub-classes (i.e. rod bipolar and ON/OFF cone bipolar cells). Although the absence of Cre immunolabeling in Type 2 OFF cells initially raised the possibility that the *Vsx2-5.3-PRE-Cre* transgene is not expressed in Type 2 bipolar cells, constitutive conditional reporter analysis suggests that this is not the case. These findings suggest that the *Vsx2-5.3-PRE-Cre* transgene is either expressed in an early bipolar cell precursor and subsequently down-regulated during Type 2 OFF differentiation, or that transgene expression in Type 2 bipolar cells is below the level of detection using immunolabeling (summarized in Figure 9A). Regardless, our data indicates that the *Vsx2-5.3-PRE-Cre* transgene represents a useful tool for targeting most if not all bipolar cells.

Despite the absence of any detectable photoreceptor Cre immunolabeling, we occasionally observed tdTomato-positive photoreceptors. This raises the possibility that the *Vsx2-5.3-PRE-Cre* transgene is active in a small population of postmitotic retinal precursors that are biased, but not fully committed, to becoming bipolar cells (Figure 9B). Interestingly, tdTomato reporter expression was not present in retinal cell types other than bipolar cells and photoreceptor cells. It has previously been shown that bipolar cell-promoting elements located in within the 1.9 kb region ∼19 kb upstream of *Vsx2* repress *Vsx2* in rod photoreceptors [15]. In addition, *Vsx2* has been shown to function in the postnatal retina as a repressor of rod photoreceptor cell fate [12]. Thus, a failed attempt at bipolar specification would be predicted to be accompanied by transient *Vsx2-5.3-PRE-Cre* transgene expression, but would ultimately result in photoreceptor production (Figure 9B). The possibility of postmitotic re-specification of retinal neurons has previously been suggested in dissociation experiments in which post-mitotic rod precursors were believed to differentiate into bipolar cells [29]. *Vsx2-5.3-PRE-Cre* transgenic mice therefore represent a useful tool to assay (using constitutive conditional reporters) changes in cell fate within bipolar/photoreceptor competent precursors in response to experimental manipulation.
